# Subsequent Migration of Immigrants Within Australia, 1981–2016

**DOI:** 10.1007/s11113-018-9482-4

**Published:** 2018-07-31

**Authors:** James Raymer, Bernard Baffour

**Affiliations:** School of Demography, Australian National University, 9 Fellows Road, Acton, ACT 2601 Australia

**Keywords:** Immigrants, Foreign-born, Internal migration, Australia

## Abstract

Australia is a major immigration country and immigrants currently represent around 28% of the total population. The aim of this research is to understand the long-term consequences of this immigration and, particularly, how migrants respond to opportunities within the country after arriving through the process of subsequent (internal) migration. The focus is on major immigrant groups in Australia, including persons born in the United Kingdom, New Zealand, China and India, and how their patterns differ from persons born in Australia. To conduct this analysis, we have gathered data for a 35-year period based on quinquennial census data. We also obtained birthplace-specific mortality data for constructing multiregional life tables for the immigrant populations. Subsequent migration is important for understanding population redistribution, and the relative attractiveness of destinations within host countries. Our results highlight the importance of subsequent migration and the diversity of migration behaviours amongst different immigrant groups in the context of overall declines in internal migration since 1981.

## Introduction

Australia is a traditional immigrant receiving country that is still growing and changing due to sustained high levels of migration. Over the past 40 years, migration to Australia has shifted primarily from the United Kingdom and Europe towards a very diverse set of origins with migrants coming from all over the world. The United Kingdom remains the dominant migrant population but this population has aged considerably and has not grown, whereas those born in New Zealand, China, India, Vietnam and elsewhere (outside of Europe) have grown considerably over the past 40 years and are relatively young.

Most migrants are concentrated in state and territory capital cities but some have settled in rural areas, motivated in part by regional immigration policies (Hugo 2008). In this paper, we are interested in whether immigrants are likely to make subsequent internal movements outside their current area of residence. In particular, we would like to know about the relative attractiveness and retention capability of areas across Australia, and whether policies that enticed migrants to reside in regional areas outside Australia’s capital cities have been effective. This information also provides insights into how immigrant groups are concentrating or dispersing across areas over the long-term, and potentially adds to our understanding of migration theory, especially in relation to social networks and cumulative causation (Massey et al. 1993). Here, the social networks of immigrants within the country may facilitate subsequent moves and, because a major migration has already been undertaken, immigrants may be more likely to consider another migration, depending on where they reside, and if there are potential benefits of doing so.

This research contributes to a small but emerging literature on the subsequent migration of immigrants in high immigration countries. Much of this work has focused on the United States and was published in the 1990s (e.g. Belanger and Rogers 1992; Frey and Liaw 1999; Kritz and Nogle 1994; Newbold 1999; Nogle 1997; Rogers and Henning 1999). More recently, the enquiries have turned towards the attractiveness and retention of non-traditional immigrant locations (Kritz et al. 2011). We are interested in how the corresponding patterns differ for Australia, where the relative share of the immigration is considerably higher and the population distribution is more concentrated. To do this work, we have gathered internal migration data disaggregated by age, sex and country-of-birth from eight Australian quenquinnial censuses from 1981 to 2016.

We focus on the following research questions. First, how important are subsequent migration flows in relation to immigration and the internal migration of the native (Australian-born) population? And, to what extent do these patterns differ across immigrant groups defined by their country-of-birth? Second, are there certain areas that exhibit high retention and destination attractiveness amongst different immigrant groups? Finally, what are the long-term implications of current migration patterns and how have these patterns changed since 1981? By answering these questions, we are able to provide a better understanding of the dynamics and diversity of internal migration in Australia, as well as the long-term tendencies of immigrant groups to settle in places that government policies have encouraged them to reside.

## Background

### Migrant Population Change in Australia

According to the Australian Bureau of Statistics, the population of Australia increased by over 60% from 15.0 million people in 1981 to 24.2 million people in 2016 (ABS 2014, 2017b). The foreign-born represented around 28% of the total population in 2016 (ABS 2017a), up from 23% 10 years earlier. This places Australia in the top twenty countries in the world with high percentages of international migrants, and second to Switzerland in the list of more developed countries with populations greater than one million (United Nations 2015). It is also one of the most ethnically diverse populations in the world (Castles et al. 2014, p. 166; Jupp 2001). With increased life expectancy and below replacement fertility occurring over the past 30 years, international migration has clearly played an important role in determining Australia’s current population size and composition (Khoo 2002; Richards 2008). What is not well understood is the extent that subsequent migration of immigrants has contributed to current spatial population sizes and distributions.

Many studies have examined the diversity of international migration in Australia (e.g. Bell and Hugo 2000; Hugo 1986; Jupp 1995; Khoo 2002, 2003; Khoo et al. 2011; Markus et al. 2009; Price 1998; Stillwell et al. 2001; Wilson and Raymer 2017), including the underlying reasons of immigration (Hugo 2004), and resulting social change (Markus et al. 2009). However, aside from Burnley (1996) and Hugo and Harris (2011), relatively little work has been undertaken regarding subsequent migration in Australia, and none have considered the influences of different countries of birth nor their trajectories over time.

The demographic study of migrant populations provides researchers and policymakers with information about the fundamental sources of change and impacts on the overall population compositions and geographic distributions. Further, as pointed out by Finney and Simpson (2008), there is a lot to learn about the population dynamics of different immigrant groups. This work thus complements and extends previous research and provides a basis for understanding the patterns of population redistribution of different immigrant groups in relation to the Australian-born population.

### Internal Migration in Australia

Internal migration has long been an important factor of population change. For example, in comparing internal migration in Australia with Great Britain, Bell et al. (2002) found that Australians have a higher propensity to migrate (i.e. double the number of moves), are less affected by distance, and exhibit patterns of migration which are more spatially concentrated. They also found that the impact of internal migration on population redistribution in Australia is greater in Britain due to their higher relative levels of migration.^1^ One of the distinctive features of internal migration over the past several decades is persistent net migration loss from New South Wales (Sydney) to other states in the country. Over 20 years ago, Burnley (1996) attributed this to high levels of immigration and housing costs. Another distinctive feature is declining propensities to make internal moves, not only in Australia (Bell et al. 2018), but in other developed countries as well (Champion et al. 2018; Cooke 2013).

The study of subsequent migration contributes to our understanding of how immigration to particular destinations leads to immigrant concentration or dispersal. Assimilation theory posits that the more established migrant populations become, the more similar they become to the native population (Glick and Park 2016). Thus, this study provides a basis for examining how various immigrant populations are converging or diverging in their internal migration patterns from the majority Australian-born population. We are particularly interested in what happens when migrants come from a very diverse set of origins and settle in different places to each other, and how their internal patterns evolve over time as their populations grow and age.

Elsewhere, studies have found that immigrants exhibit higher propensities of internal migration than native-born populations (Belanger and Rogers 1992; Reher and Silvestre 2009; U.S. Census Bureau 2003). As a result, subsequent migration of immigrants can represent an important component of subnational population change and may even exceed the migration component from abroad (Nogle 1997). Moreover, subsequent migration patterns of immigrants are linked to the spatial distribution of their compatriots (Kritz and Nogle 1994). That is, migrants are less likely to move away from areas with high concentrations of immigrants and more likely to move towards them. Kritz et al. (2011) show that out-migration of the foreign-born from cities in the United States are motivated by both employment opportunities and network factors. However, the levels and spatial patterns vary across birthplace origins and levels of education or skill (see also Reher and Silvestre 2009). This is supported by Newbold (1999), who found that subsequent migration acted to further concentrate foreign-born populations for some birthplace groups, while dispersing them in others (see also U.S. Census Bureau 2003). In studying internal migration in Canada, on the other hand, Newbold (1996) found foreign-born populations responded to economic opportunities in similar ways as the native-born population with most of the differences explained by individual characteristics rather than the country-of-origin.

When examining Australia’s internal migration, one factor to consider is the high degree of population concentration in the state capital cities. For immigrant populations, Sydney and Melbourne are particularly dominant (Hugo 2008). This may result in distinct life course mechanisms in relation to other countries. For example, Bernard et al. (2016) found that the movements of people in Australia were less affected by transitions to higher education, entry into the labour force, partnering and family formation than, for example, Great Britain where the distances moved tended to be much shorter.

There have also been studies on the possible displacement impacts of immigration on native-born populations with varied results. For example, Burnley (1996) and Frey (1996; see also Frey and Liaw 1998, 1999) found strong associations between areas of high immigration and areas of negative net migration of low-skilled native-born internal migrants. Wright et al. (1997), on the other hand, found that once you control for population size, foreign-born and native-born migrants respond in similar ways to various opportunities. These variations in research findings on the effects of immigration on native internal migration are thought to be the result from differences in sample design, methodological approaches and construction of comparable skill groups (Borjas 2014, p.131). Although not the focus of this research, it is worthwhile to understand the implications of population changes resulting from both immigration and internal migration, and how these changes may be associated with the internal migration of the native population.

## Data and Methods

Internal migration and immigration data have been gathered from the Australian quinquennial censuses from 1981 to 2016. They represent data for 19 countries of birth, including the Australian-born population, across 47 geographic areas. Similar to Kritz et al. (2011), we are interested in the heterogeneity amongst migrant groups and different immigrant destinations, both traditional and otherwise. The 19 birthplace-specific populations and their sizes in 2016 are presented in Table 1. The Australian-born population represented about 72% of the total population. The remaining 28% are spread across 18 populations with the largest numbers of persons born in the United Kingdom, South-East Europe, New Zealand, China and India.

**Table 1:** Populations in Australia by country or region of birth, 2016 *Source* Australian Bureau of Statistics, Estimated Resident Populations

	Birthplace	Population	% total foreign-born
1	Australia	17,254,110	
2	New Zealand	607,230	8.8
3	Other Oceania and Antarctica	165,370	2.4
4	United Kingdom	1,197,970	17.4
5	Other North-West Europe	414,080	6.0
6	South-East Europe	788,820	11.5
7	North Africa and the Middle East	410,320	6.0
8	Vietnam	236,750	3.4
9	Philippines	246,430	3.6
10	Malaysia	166,150	2.4
11	Indonesia	83,780	1.2
12	Other South-East Asia	240,000	3.5
13	China (excludes SARs, Taiwan)	526,040	7.7
14	Other North-East Asia	326,900	4.8
15	India	468,830	6.8
16	Other Southern and Central Asia	334,400	4.9
17	North America	181,500	2.6
18	South America	123,660	1.8
19	Sub-Saharan Africa	354,560	5.2

**Fig. 1:**
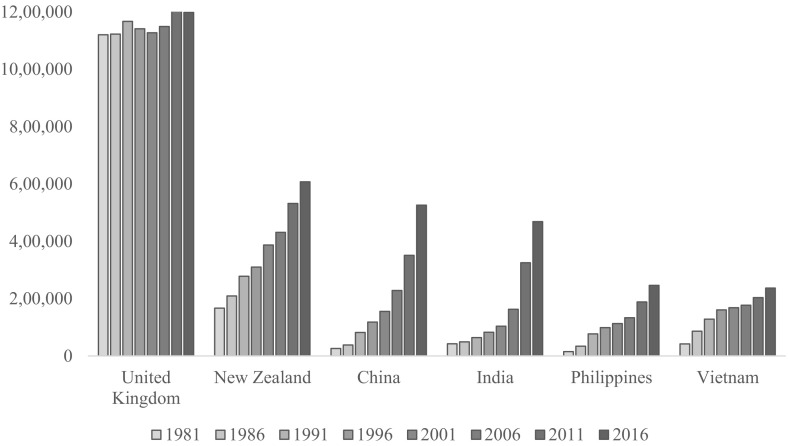
Population change for the six largest immigrant groups in Australia, 1981–2016

In terms of changes in population sizes over time, the six largest migrant populations in Australia (measured in 2016) are presented in Fig. 1. Here, we see that the migrant population born in the United Kingdom is the largest but has not changed much in size since 1981. The other migrant populations have grown with China and India showing especially rapid growth in recent years.

The birthplace-specific internal migration data were harmonised for 47 geographic areas from 1981 to 2016 (Baffour and Raymer 2018). These data were commissioned from the Australian Bureau of Statistics that provided data based on the Statistical Division geography from Australian Standard Geographical Classification. Note that the geographic boundaries of the Statistical Division geographies are not consistent over time. For any given year, there are around 60 Statistical Divisions. In 2016, however, the Australian Bureau of Statistics ceased releasing tabulations based on Statistical Divisions. The closest geography available was the SA4 geography, which contained 88 areas but with substantially different geographic boundaries than the Statistical Division geography. Therefore, we utilised the SA3 geographic areas, representing 358 spatial units, and converted them to approximate the 47 harmonised Statistical Divisions (see below). Blake et al. (2000) developed a procedure for harmonising areas across Statistical Divisions in Australia but we were unable to utilise it for two reasons. First, they had access to a lower level of geography, which we did not have access to. As our data were considerably more detailed, the costs for commissioning data at a lower level of geography from 1981 to 2016 were too prohibitive. Second, there were no available correspondence tables for the differing geographic boundaries after 1996.

**Fig. 2:**
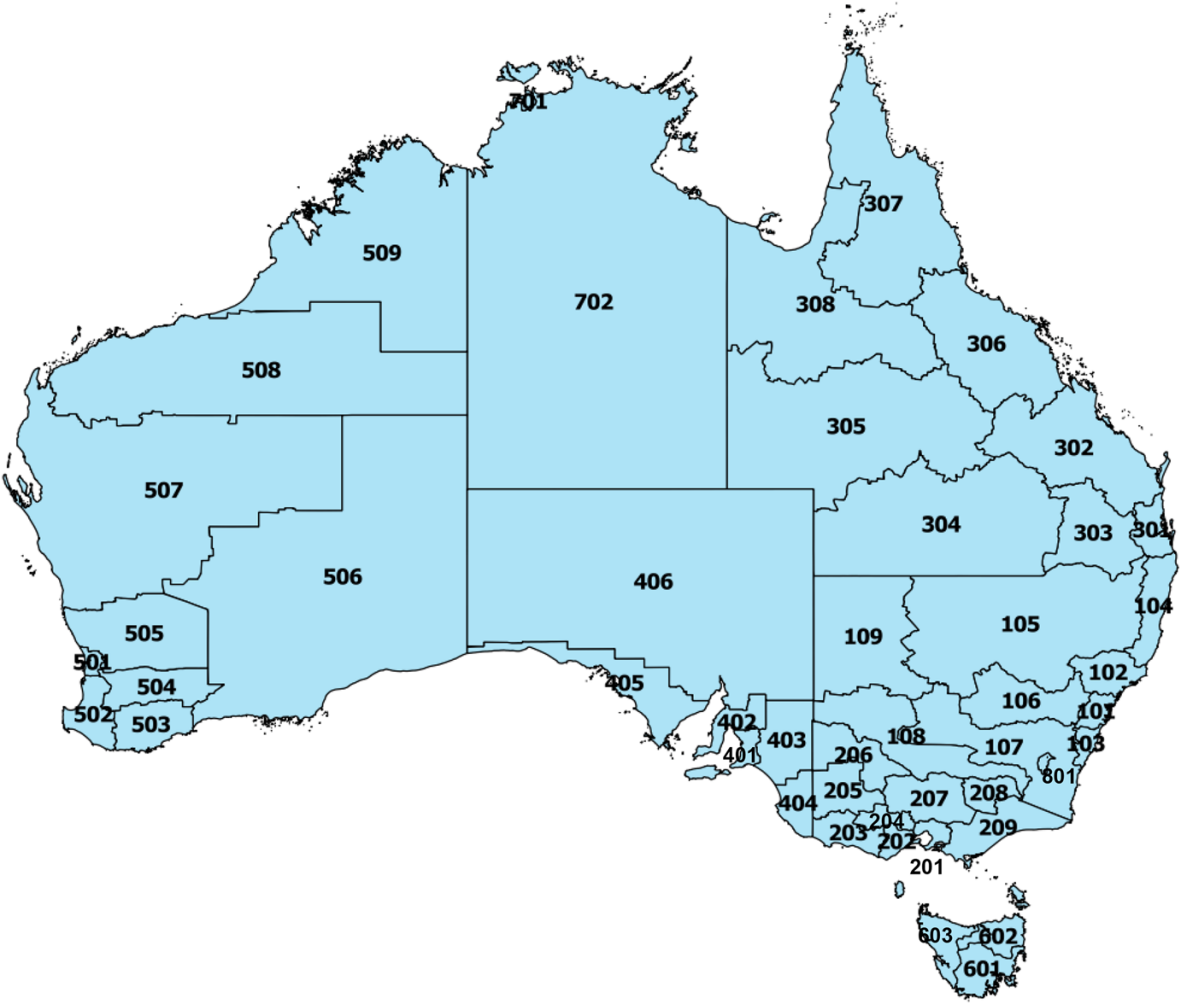
Map of consistent geographic areas between 1981 and 2016 census years for Australia. *Note* area names are listed in Appendix 1

To produce a consistent geography over time, we were forced to use simple rules that either assumed the boundary changes were insubstantial (if the boundary change resulted in only a small amount of population change) or merged multiple geographic areas into single (larger) ones. These rules worked reasonably well and produced a meaningful geography for studying subsequent migration of immigrant populations across 47 areas. The only geographic area that required additional input was Darwin in the Northern Territory. Here, the population size was altered to correspond to the geographic area change. A map of the 47 areas used for the analysis is presented in Fig. 2, and the corresponding list of area names is included in Appendix 1.

In addition to collecting data from censuses on internal migration and immigration, we also gathered birthplace-specific mortality data and used models to smooth the sparse data across the 47 areas (Baffour and Raymer 2018). This was needed in order to calculate multiregional life tables. Multiregional life tables (Rogers 1975, 1995) are useful for understanding the potential long-term consequences of internal migration. They provide measures on the expected duration of years to be spent in each area, given their place of origin. They also can be used to calculate retention expectancies for each area, which for a given exact age, can be used to measure an area’s ability to retain migrants over time.

## Levels and Relative Shares of Subsequent Migration

In this section, we analyse the overall levels of internal migration and the relative shares of in-migration and out-migration to each area covering the five-year periods 1981–1986 to 2011–2016. The overall level of migration is important for understanding the changes that are mostly related to population growth and the intensity of migration. For space reasons, we only present the calculations for the largest country-of-birth populations.

The levels of internal migration for persons born in Australia, United Kingdom, New Zealand, China, India, Vietnam and the Philippines are presented in Table 2A. It is clear that the largest internal migration flows are comprised by those born in Australia, which is not surprising considering they represent the majority population. The next largest flows, again not surprising, are made up of persons born in the United Kingdom and New Zealand, which represent the two largest countries of birth for migrants in Australia (see Table 1). The patterns for China, India, Vietnam and the Philippines, however, are less clear. For example, although the Chinese-born population is larger than the Indian-born population, the subsequent migration flows are smaller. Also, while the subsequent migration flows for persons born in China, India and the Philippines have increased over time, the same is not the case for those born in Vietnam. Further, for migrants born in the United Kingdom, the subsequent migration levels have dropped considerably from 112 thousand in 1981–1986 to 58 thousand in 2011–2016 even though the population size remained about the same—a likely consequence of the population ageing that occurred during this time (Wilson and Raymer 2017).

**Table 2:** Overall levels of migration within and to Australia by selected countries of birth, 1981–1986 to 2011–2016 *Source* 1981–2016 Australian Censuses

Period	Australia	United Kingdom	New Zealand	China	India	Vietnam	Philippines
A. Internal migration							
1981–1986	1,322,680	112,299	23,081	1389	2973	3718	1121
1986–1991	1,410,662	115,932	29,712	1731	3291	4889	2870
1991–1996	1,373,678	100,081	31,214	3709	3767	5029	4073
1996–2001	1,372,480	88,770	31,985	3744	4573	5002	4313
2001–2006	1,288,653	80,588	34,345	4282	5194	4254	4575
2006–2011	1,212,234	69,900	36,081	7793	11,654	3483	5438
2011–2016	1,250,898	58,063	29,814	13,448	22,913	2899	6037
B. Stayers (persons who did not migrate)							
1981–1986	9,402,014	841,834	104,932	23,135	34,334	39,060	14,525
1986–1991	9,817,075	853,997	139,502	35,807	40,386	78,862	32,503
1991–1996	10,266,596	870,308	188,373	70,031	51,914	115,233	65,090
1996–2001	10,780,390	847,623	214,550	94,438	63,500	132,614	78,896
2001–2006	11,095,304	814,975	257,379	127,367	80,675	138,128	90,937
2006–2011	11,969,586	858,956	308,720	184,624	137,721	153,555	113,641
2011–2016	12,470,864	871,324	363,416	280,965	258,925	175,927	158,352
C. Immigration							
1981–1986	79,429	99,051	62,489	10,938	8696	36,032	15,328
1986–1991	65,013	102,436	77,965	28,895	13,358	25,396	27,356
1991–1996	85,312	68,250	53,341	31,152	18,546	20,698	18,347
1996–2001	95,839	71,617	87,757	38,262	23,562	9773	15,982
2001–2006	116,558	106,758	73,996	65,251	53,473	10,331	19,433
2006–2011	129,528	134,071	109,831	113,094	129,040	20,589	44,307
2011–2016	127,324	104,843	86,142	197,247	148,245	30,558	54,276
D. Proportion of internal migration (A/A + B)							
1981–1986	0.12	0.12	0.18	0.06	0.08	0.09	0.07
1986–1991	0.13	0.12	0.18	0.05	0.08	0.06	0.08
1991–1996	0.12	0.10	0.14	0.05	0.07	0.04	0.06
1996–2001	0.11	0.09	0.13	0.04	0.07	0.04	0.05
2001–2006	0.10	0.09	0.12	0.03	0.06	0.03	0.05
2006–2011	0.09	0.08	0.10	0.04	0.08	0.02	0.05
2011–2016	0.09	0.06	0.08	0.05	0.08	0.02	0.04
E. Ratio of internal migration to immigration (A/C)							
1981–1986	16.65	1.13	0.37	0.13	0.34	0.10	0.07
1986–1991	21.70	1.13	0.38	0.06	0.25	0.19	0.10
1991–1996	16.10	1.47	0.59	0.12	0.20	0.24	0.22
1996–2001	14.32	1.24	0.36	0.10	0.19	0.51	0.27
2001–2006	11.06	0.75	0.46	0.07	0.10	0.41	0.24
2006–2011	9.36	0.52	0.33	0.07	0.09	0.17	0.12
2011–2016	9.82	0.55	0.35	0.07	0.15	0.09	0.11

Immigration of Australian-born = returning nationals

In Table 2D, the proportion of subsequent migration across all areas is presented for the same seven populations as above. These measures are calculated by dividing the internal migration flows (Table 2A) by the population at the beginning of the time interval (Table 2A + B). From these proportions, we see that the Australian-born and United Kingdom-born populations have high levels of internal migration ranging from 12–13% in 1986–1991 to 6–9% in 2011–2016. The New Zealand-born migration proportions are even higher, ranging from 18% in 1981–1986 to 8% 2011–2016. In all three of these cases, the levels of migration were higher in the 1980s than they were in the more recent periods. For the migrant populations born in China, India, Vietnam and the Philippines, the proportions of internal migration are much lower ranging from 9% (Vietnam, 1981–1986) to 2% (Vietnam, 2011–2016). Of these four immigrant groups, India tended to have the highest levels of subsequent migration. Finally, the proportions of internal migration decreased over time for all population groups.

Ratios of internal migration to immigration are presented in Table 2E. These ratios capture the relative importance of internal migration to immigration. For the Australian-born population, internal migration is several factors larger in size than the numbers of returning from abroad. For example, during the period 1986–1991, there were nearly 22 times more internal migrants than there were returning nationals. This ratio decreased to just over 9 during the 2006–2011 and 2011–2016 periods. During the 1980s and 1990s, the internal migration to immigration ratios for the United Kingdom-born population ranged from 1.1 to 1.5, but then dropped to 0.5 during 2006–2011 and 2011–2016. For the other immigrant groups, immigration flows were much larger than internal migration flows, especially for the Chinese-born population where the level of internal migration during the 2011–2016 period represented only about 7% of the immigration flows.

**Fig. 3:**
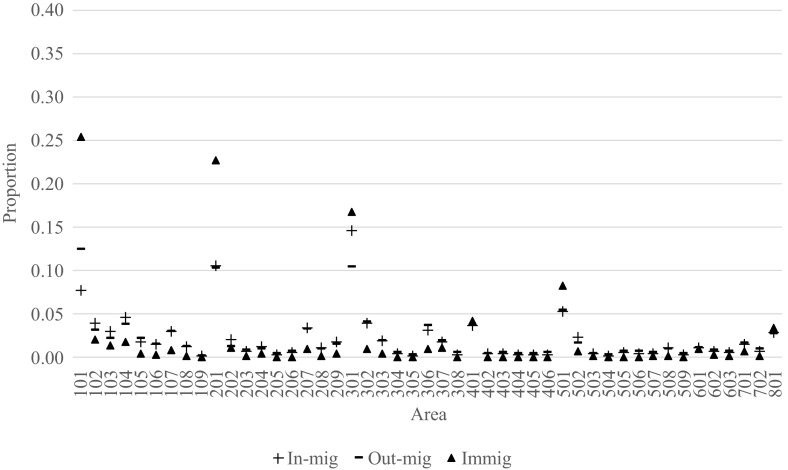
Relative shares of in-migration, out-migration and immigration to each area in Australia for persons born in Australia, 2011–2016

**Fig. 4:**
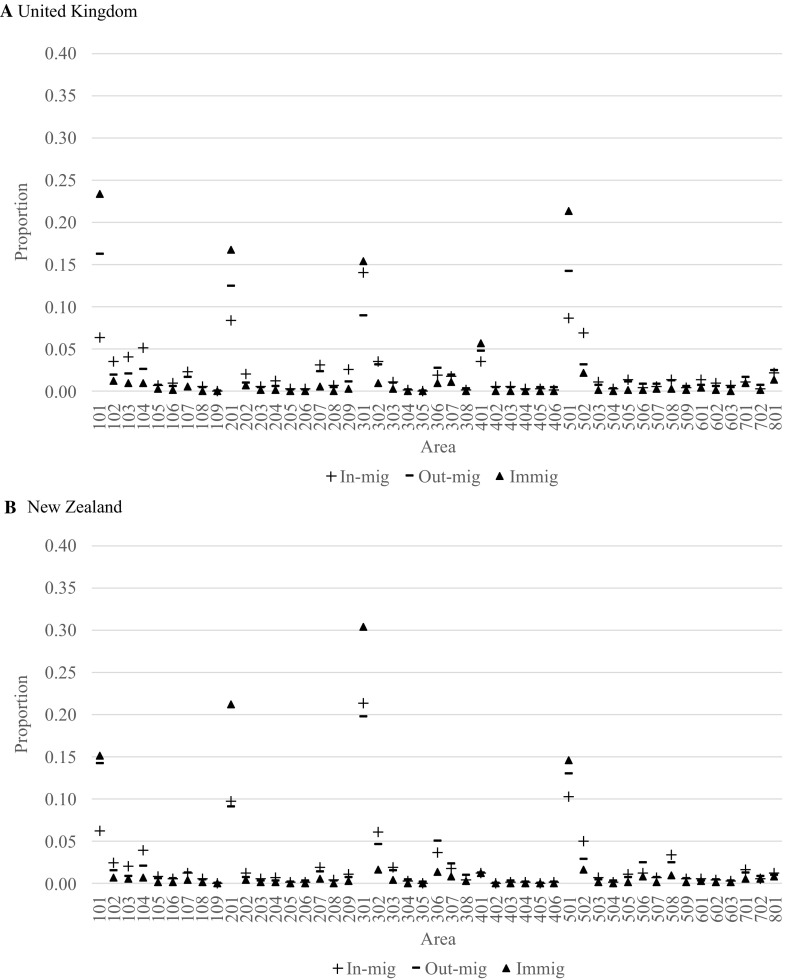
Relative shares of in-migration, out-migration and immigration to each area in Australia for persons born in United Kingdom and New Zealand, 2011–2016

**Fig. 5:**
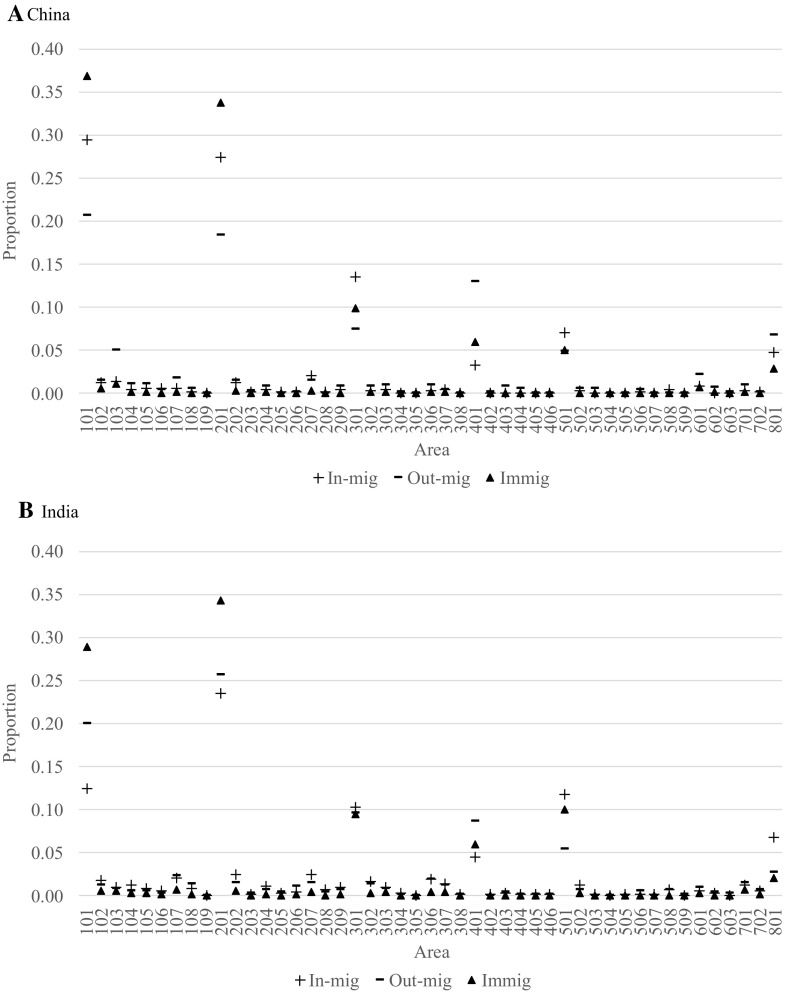
Relative shares of in-migration, out-migration and immigration to each area in Australia for persons born in China and India, 2011–2016

In addition to the overall levels of internal migration, the 2011–2016 proportions of total internal migration to and from each area, along with the corresponding proportions of immigration, are presented in Figs. 3, 4, and 5 for the populations born in Australia, United Kingdom, New Zealand, China and India. As a basis for the comparisons, the Australian-born patterns are shown first in Fig. 3. Here, we see that the largest proportions of in-migration, out-migration and immigration are to be found in the capital city areas of Sydney (101), Melbourne (201), Brisbane (301), Adelaide (401), Perth (501) and Canberra (801). The exceptions are Greater Hobart (601) and Darwin (701). Refer to Appendix 1 for correspondence between area codes and area names.

Amongst the capital city areas, there are substantial variations in the relative shares of in-migration, out-migration and immigration amongst the Australian-born population (Fig. 3). Relative to internal migration, immigration is more concentrated towards Sydney (25%), Melbourne (23%), Brisbane (17%) and Perth (8%). Together, these four areas receive nearly three quarters of all Australian-born returning from abroad. For the remaining 35 areas, immigration of Australian-born comprises a relatively smaller share than in-migration and out-migration. Furthermore, most areas send and receive around the same share of in-migration and out-migration. However, Sydney stands out as it sends a substantially larger share of migrants than it receives (12 and 8%, respectively), whereas the opposite is the case for Brisbane (10 and 15%, respectively).

In Fig. 4, the relative shares of in-migration, out-migration and immigration are presented for the migrants born in the United Kingdom and New Zealand. Here, we see that Perth is especially attractive for migrants born in the United Kingdom, just below the level of Sydney and above Brisbane and Melbourne. For migrants born in New Zealand, it is Brisbane that clearly stands out as the main destination receiving over 30% of all immigrants. For the United Kingdom-born population, there are substantial losses due to internal movements for Sydney, Melbourne, Adelaide and Perth, with gains pretty much everywhere else. For the New Zealand-born population, the internal migration losses are only noticeable for Sydney and Perth.

As another illustration, consider the shares of in-migration, out-migration and immigration for China and India in Fig. 5. For both of these migrant populations, immigration to Sydney and Melbourne stands out receiving 71 and 63%, respectively, of total Chinese-born and Indian-born immigration, respectively. For the Chinese-born population, Sydney is the preferred destination, whereas for the Indian-born population, it is Melbourne. The largest gains of Chinese-born internal migrants are found in Sydney, Melbourne, Brisbane and Perth. These patterns are strikingly different from the patterns exhibited by the Australian-born, United Kingdom-born and New Zealand-born populations. Amongst the Indian-born subnational populations, Perth and Canberra receive relatively larger shares of in-migration, whereas Sydney, Melbourne and Adelaide produce relatively larger shares of out-migration.

To summarise, the levels of migration described in this section show the dominance of the capital city areas across all populations. They also show that even amongst the largest populations, there are major differences in the patterns and relative importance of subsequent migration. Some immigrant groups, such as those born in New Zealand, have relatively high propensities for internal migration, whereas others, such as those born in China have relatively low propensities of internal migration.

## Area Retention and Attractiveness

To understand the underlying patterns of subsequent migration, it is important to examine the retention and attractiveness of areas within a country. Retention refers to an area’s ability to prevent migrants from moving elsewhere. Attractiveness, on the other hand, refers to an area’s relative appeal to migrants coming from elsewhere in the country. Of course, the two processes are related and it might be expected that areas with high retention are also those that are attractive to migrants from other places. In Australia, the study of retention and attractiveness is needed to assess policies designed to distribute international migrants towards areas outside capital cities (Hugo 2008).

### Out-Migration Propensities

**Fig. 6:**
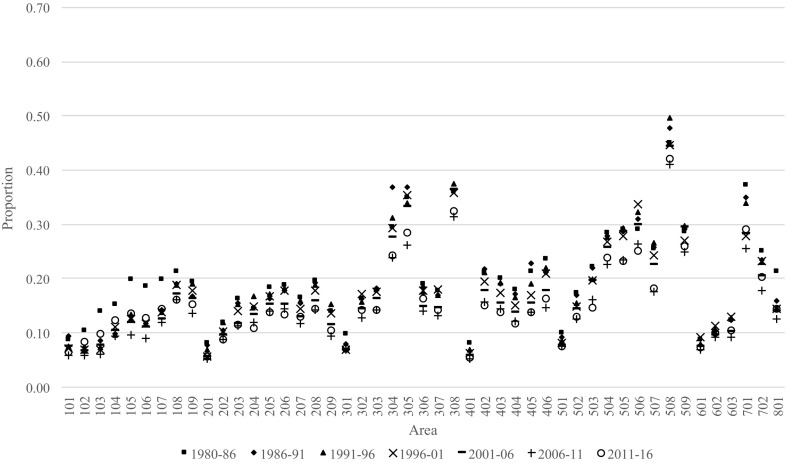
Out-migration proportions from each area in Australia for persons born in Australia, 1980–1986 to 2011–2016

In this section, out-migration proportions are used as measures of retention, where an area with high levels of out-migration can be said to have a low retention propensity. As a basis for understanding the patterns, consider the Australian-born out-migration proportions presented in Fig. 6, which covers the periods 1981–1986 to 2011–2016. The first thing to notice is that the capital city areas (i.e. 101, 201, 301, …, 801) tend to have low out-migration proportions and, thus, high retention. These patterns have been relatively stable over time, albeit with a trend towards lower out-migration proportions over time across all areas. Outside capital city areas, we find much higher and more variable out-migration proportions. The highest out-migration proportions are found in Pilbara (508)—an area in Western Australia whose main industry is mining petroleum, natural gas and iron ore deposits—ranging from just over 40% in 2006–2011 to just under 50% 1991–1996. This implies that nearly half the population who were living in this area moved elsewhere within a five-year period and these very high levels have been maintained since 1981. Other notable areas with high levels of out-migration include South West Queensland (304), Central-West Queensland (305), North-West (308), South-Eastern WA (506) and Darwin (701).

**Fig. 7:**
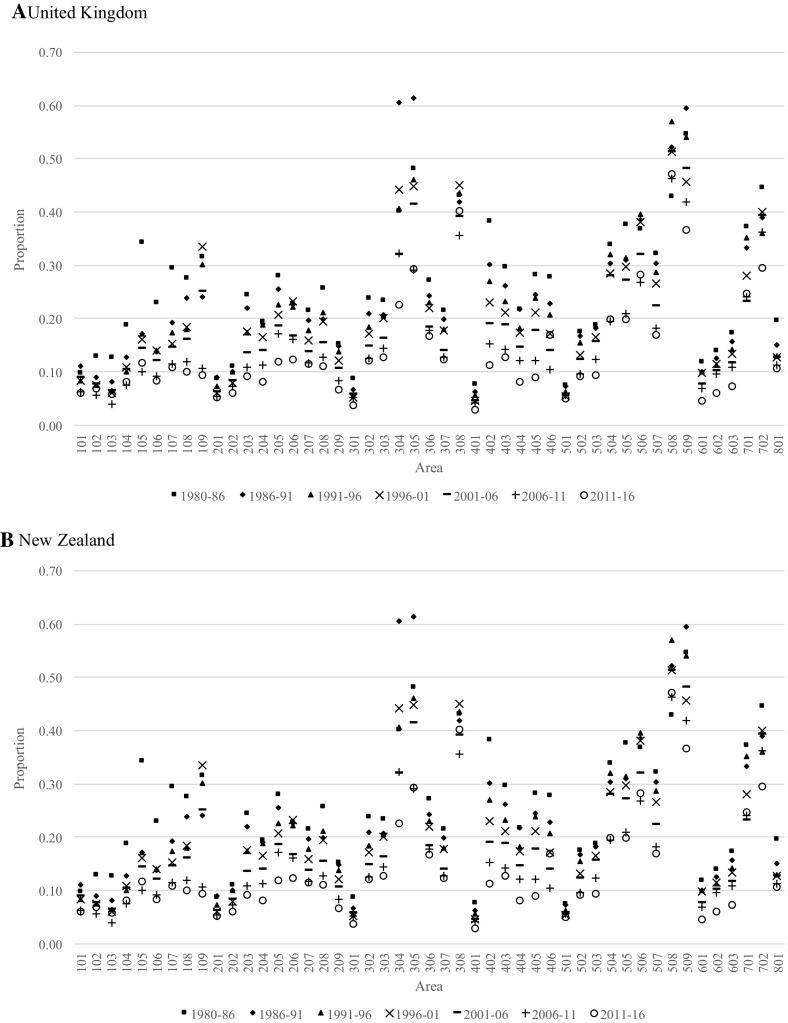
Out-migration proportions from each area in Australia for persons born in the United Kingdom and New Zealand, 1980–1986 to 2011–2016

**Fig. 8:**
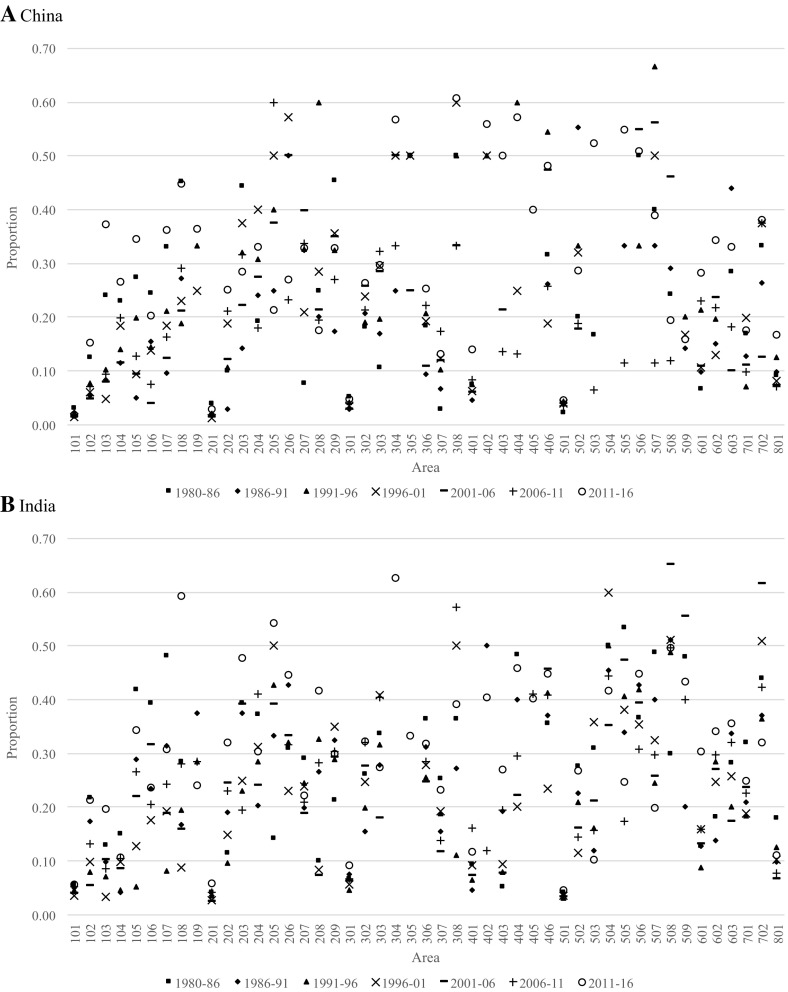
Out-migration proportions from each area in Australia for persons born in China and India, 1980–1986 to 2011–2016. *Note* Proportions affected by sparse data; zeros and proportions exceeding 0.7 excluded

As in the previous section, we compare the patterns of the Australian-born population with the subsequent migration patterns of those born in the United Kingdom and New Zealand (Fig. 7), representing large and established migrant populations, and China and India (Fig. 8), representing large and more recent migrant populations. The main difference between the migrant populations and the Australian-born is the higher out-migration proportions from non-capital city areas and more variability. Also, while the subsequent out-migration proportions for Chinese-born and Indian-born proportions are extremely variable due to their very small population sizes in regional areas, they appear to be increasing over time. This is different for capital cities, where the proportions have remained remarkably low and consistent over time—especially in Sydney, Melbourne, Brisbane and Perth.

### Multiregional Life Expectancies

In order to further understand the retention and attractiveness of areas across Australia, we calculated multiregional life expectancies (Rogers 1975, 1995), which express the number of remaining years of life that one can expect to live in a particular area at a given age—assuming they remain in Australia for the remainder of their lives. For comparison purposes, the remaining years of life have been converted into percentage of remaining life.

**Fig. 9:**
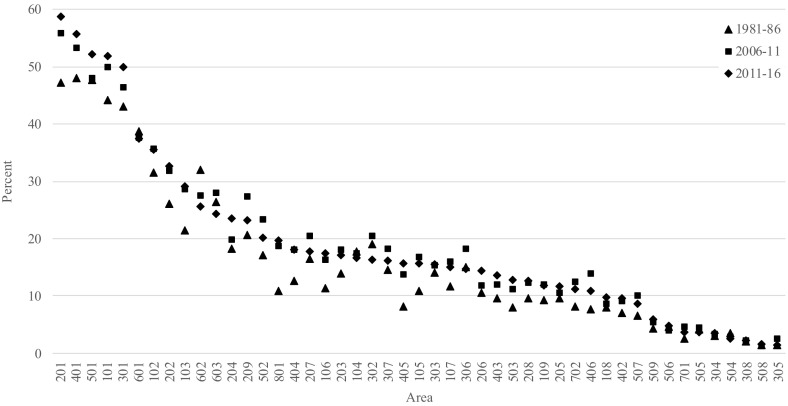
Area retention expectancies (percent) at age 25 years for females born in Australia, 1981–1986, 2006–2011 and 2011–2016

In Fig. 9, the area retention expectancies at age 25 years are presented for females born in Australia for three periods: 1981–1986, 2006–2011 and 2011–2016. These represent the percentage of remaining life that can be expected to be spent in their current area of residence. The area with the highest retention expectancy during the two most recent periods is Melbourne with 56 and 59%, respectively—that is, on average, 25 year olds could expect to live at least 56% of their remaining life in the same area based the mortality and migration patterns of the 2006–2011 period, which then increased by another three percent during the 2011–2016 period. The lowest retention expectancies during the 2011–2016 period are found in Pilbara (Western Australia), where only about 2% of remaining years can be expected to be spent in the same area.

Between the 1981–1986, 2006–2011 and 2011–2016 periods, the retention patterns of Australian-born remained fairly stable (Fig. 9). In all three periods, the capital city areas and their neighbouring areas exhibited the highest retention expectancies. These increased noticeably for Melbourne, Adelaide, Perth, Sydney, Illawarra and Canberra. The only area that exhibited substantial decrease in the retention expectancy was Northern Tasmania (602).

**Fig. 10:**
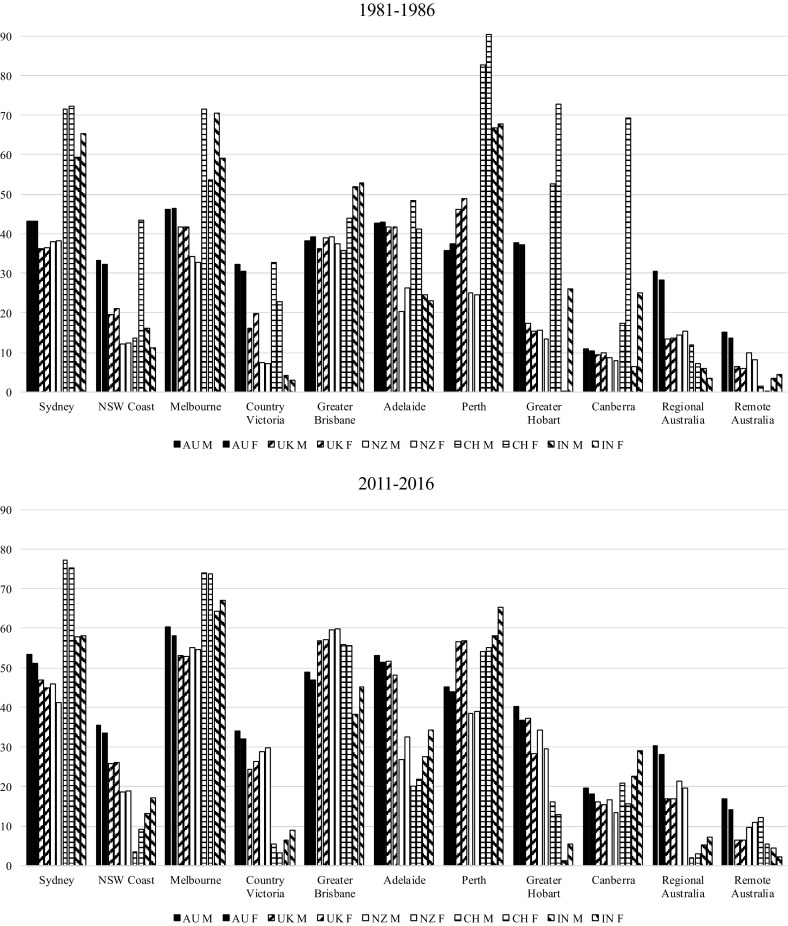
Male and female area retention expectancies (percent) at age 25 years for persons born in Australia, United Kingdom, New Zealand, China and India: 1981–1986 and 2011–2016. *Note* Sydney = 101; NSW Coast = 102, 103 and 104; Melbourne = 201; Country Victoria = 202–204, 207 and 209; Greater Brisbane = 301; Adelaide = 401; Perth = 501; Greater Hobart = 601; Canberra = 801; Regional Australia = 105–108, 205, 208, 302–303, 306, 402–404, 502–503, 602 and 701; and Remote Australia = 304–305, 307–308, 405, 506–508, 603 and 702 (see Appendix 1 for area names)

In Fig. 10, the 1981–1986 and 2011–2016 male and female retention expectancies at age 25 years for the Australian-born population are compared with those born in the United Kingdom, New Zealand, China and India for different areas in Australia. Note, because of the sparseness in the regional data for nearly all immigrant groups, we focus on the large capital cities and their surrounding areas and group the remainder into Regional Australia and Remote Australia. With these aggregations, some caution remains for interpreting the 1981–1986 patterns due to some relatively small numbers underlying the calculations (e.g. Chinese-born females in New South Wales (NSW) Coast and Canberra).

For the 2011–2016 data presented in Fig. 10, migrants born in China and India exhibited higher retention expectancies in Sydney and Melbourne but around the same or lower elsewhere. Migrants born in New Zealand exhibited the highest retention values for Brisbane. Persons born in Australia, the United Kingdom and New Zealand had much higher retention expectancies in NSW Coast, Country Victoria, Greater Hobart and Regional Australia in comparison to the two other population groups. The patterns for Adelaide was similar but persons born in New Zealand exhibited about the same levels of retention as those born in India.

To study area attractiveness, multiregional life expectancies for females at age 25 years are presented in Table 3 for six origin areas (note, the results for males were similar but are not included for space reasons). These numbers represent the percentage of remaining life that can be expected to be lived amongst the eleven areas given the area of residence at age 25 years. These calculations are based on the 2011–2016 migration and mortality data. We have excluded the five largest capital city origin areas of Sydney, Melbourne, Greater Brisbane, Adelaide and Perth to focus on the less populated origin areas. Our main interest here is the percentage of remaining life that can be expected to be spent in other areas provided the population remains in Australia (i.e. does not emigrate). We are also interested in how populations born in the United Kingdom, New Zealand, China and India differ from each other and from the Australian-born.

**Table 3:** Multiregional life expectancies (percent) for females aged 25 years born in Australia, United Kingdom, New Zealand, China and India, 2011–2016

Origin	Birth	Destination										
		Sydney	NSW Coast	Melbourne	Country Victoria	Greater Brisbane	Adelaide	Perth	Greater Hobart	Canberra	Regional Australia	Remote Australia
NSW Coast	Aus.	18.4	**33.6**	7.1	2.2	15.9	2.2	3.1	0.6	2.2	12.7	2.1
	UK	20.1	**26.0**	8.9	2.2	25.9	1.3	7.5	0.2	2.5	4.9	0.6
	NZ	18.2	**18.8**	13.0	1.4	30.1	0.7	6.5	0.2	1.7	8.1	1.3
	China	59.4	**9.1**	13.3	0.2	11.1	1.4	2.3	0.1	2.9	0.1	0.0
	India	38.9	**17.0**	17.3	0.4	12.3	1.1	6.3	0.0	2.6	4.0	0.1
Country Victoria	Aus.	3.8	2.9	34.3	**32.0**	8.3	2.4	3.1	0.6	1.1	9.7	1.9
	UK	5.7	0.9	46.1	**26.3**	10.6	1.1	4.5	0.2	0.9	3.4	0.2
	NZ	4.3	0.7	40.5	**29.8**	12.9	0.5	6.4	0.4	0.8	3.1	0.6
	China	13.2	0.5	70.3	**3.3**	5.1	1.7	3.9	0.3	1.4	0.2	0.1
	India	7.6	0.4	67.6	**9.0**	4.7	1.5	5.3	0.1	1.5	2.2	0.1
Greater Hobart	Aus.	6.1	3.6	14.5	3.7	11.9	3.0	4.5	**36.7**	1.7	10.8	3.4
	UK	6.1	1.6	20.2	4.3	16.9	1.9	13.6	**28.4**	0.9	5.3	0.9
	NZ	5.0	0.8	26.1	1.5	20.8	0.5	9.7	**29.6**	0.4	4.3	1.4
	China	21.0	0.7	44.6	0.7	12.3	1.4	2.6	**13.0**	3.1	0.4	0.1
	India	9.4	2.2	47.8	1.1	11.7	4.9	12.6	**5.3**	1.6	3.3	0.1
Canberra	Aus.	14.4	7.7	13.5	3.6	15.2	3.7	4.6	0.9	**18.0**	16.2	2.3
	UK	16.8	5.2	12.9	2.0	21.2	3.9	10.6	0.2	**15.3**	11.0	0.7
	NZ	13.3	4.3	17.0	4.4	26.9	2.7	8.0	0.3	**13.3**	8.2	1.6
	China	38.0	1.4	29.3	0.5	9.2	2.5	3.0	0.1	**15.7**	0.3	0.1
	India	24.5	0.6	22.0	0.5	10.0	2.1	9.0	0.0	**29.1**	2.1	0.0
Regional Australia	Aus.	8.9	7.6	10.3	4.7	19.7	5.3	7.7	1.0	2.6	**28.0**	4.1
	UK	9.0	3.9	10.2	1.4	28.0	4.4	22.0	0.4	2.3	**16.9**	1.4
	NZ	8.0	2.8	9.8	1.7	38.0	1.4	13.8	0.4	1.2	**19.5**	3.4
	China	32.3	1.5	22.2	0.3	23.2	2.8	10.2	1.1	3.0	**2.8**	0.6
	India	15.4	0.8	31.5	1.4	18.5	4.7	14.9	0.1	5.4	**7.2**	0.2
Remote Australia	Aus.	6.4	5.5	9.3	3.8	17.8	8.2	11.9	1.7	1.6	19.8	**14.0**
	UK	7.2	2.2	9.6	1.8	25.5	6.9	28.3	0.6	1.3	10.2	**6.5**
	NZ	6.7	1.9	8.9	1.2	33.8	1.8	21.6	0.8	0.5	12.0	**10.9**
	China	17.6	1.5	21.6	0.3	24.5	2.2	24.9	0.1	1.6	0.3	**5.5**
	India	8.5	0.4	25.2	0.7	20.0	4.7	31.8	0.1	2.1	4.3	**2.2**

Sydney = 101; NSW Coast = 102, 103 and 104; Melbourne = 201; Country Victoria = 202–204, 207 and 209; Greater Brisbane = 301; Adelaide = 401; Perth = 501; Greater Hobart = 601; Canberra = 801; Regional Australia = 105–108, 205, 208, 302–303, 306, 402–404, 502–503, 602 and 701; and Remote Australia = 304–305, 307–308, 405, 506–508, 603 and 702 (see Appendix 1 for area names). The bold values indicate percentage life expectancy to remain in the same area

Consider, for example, the females originating in NSW Coast. The main destinations outside this area for females born in Australia and the United Kingdom are Sydney and Greater Brisbane (and Regional Australia for the Australian-born). For those born in New Zealand, Greater Brisbane is the main area of attraction. That is, a 25 year old female New Zealander living in NSW Coast can expect to spend about 30% of her remaining years in Greater Brisbane, which is nearly twice the expectation for remaining in NSW Coast. Female immigrants born in China or India will almost certainly move to Sydney from NSW Coast. Both population groups have very low likelihoods of remaining in NSW Coast. As another example, consider females originating in Regional Australia. Here, all five populations had a strong preference for Greater Brisbane with the Chinese-born females also going to Sydney and Melbourne. The same is true for Indian-born females with the addition of Perth as an attractive destination.

Overall, in Table 3, we find several important differences in the attractiveness of areas in Australia amongst the five birthplace populations. First, the patterns of migrants born in China and India are distinctive from the patterns of persons born in Australia, United Kingdom and New Zealand. Not only do they have lower retention percentages (except Canberra), they have strong preferences for Sydney and Melbourne as a subsequent destination. Second, migrants from New Zealand have a clear preference for Greater Brisbane as a subsequent destination. Third, migrants from the United Kingdom are fairly similar to those for the Australian-born population.

## Conclusion

The study of subsequent migration is needed for understanding the places that migrants are ultimately attracted towards for employment or building their communities. Our analysis of Australian data found that immigrant groups differed in their subsequent migration patterns with some having relatively high levels of internal migration (e.g. Indian-born) and others with relatively low levels (e.g. Chinese-born). Similar to previous studies focusing on the United States and Canada, we found that subsequent migration is an important aspect of the immigrant settlement process but that it works in different ways depending on the country or region of origin (Newbold 1999; Nogle 1997; Reher and Silvestre 2009). Future research is needed to explore the mechanisms underlying these differences. Here, the social support versus economic opportunities framework provided by Kritz et al. (2011) provides a useful starting point for investigating the key factors likely to be influencing subsequent migration differences amongst immigrant groups.

Our study finds strong stability in the subsequent migration system over time but with steady declines in the overall propensities of internal migration since the 1981–1986 period. This reflects a general trend across most groups, including the Australian-born population. Studying why this is occurring and the implications for regional development and society should be a high priority for the Australian government. The reasons behind these patterns are likely to be related to ageing and changes to the economic system from manufacturing to service industries (Kritz et al. 2011). It could also be related to the rapidly changing composition of the immigrant population in which two processes are occurring: (1) the more established migrant groups from the United Kingdom and rest of Europe are rapidly ageing; and (2) migrant populations from elsewhere in the world are concentrated in the young adult age groups and are rapidly growing. Moreover, the more recent migrant groups in Australia exhibit lower levels of subsequent migration than did the earlier European immigrant groups.

Sydney, Melbourne and Brisbane, and to a lesser extent, Adelaide and Perth, stand out in terms of their importance to the internal migration system in Australia. Our research demonstrated strong persistence over time in the high levels of out-migration from regional and remote areas. State capital city areas, especially Sydney and Melbourne, on the other hand, have not only attracted both immigrants and internal migrants, but they have increasingly retained them over time. The patterns for newer immigrant groups are exhibiting even more concentrated migration patterns towards capital city areas. Thus, policies attempting to direct immigrants to regional locations outside capital city areas need to provide, in addition to employment, a range of social, economic and educational opportunities. Indeed, we found the proportions of out-migration from regional areas to be increasing over time for the Chinese-born and Indian-born populations. Without such provisions, regional and remote areas of Australia will continue struggling to retain their populations, as they have for the past 35 years.

The analysis contained in this paper focused primarily on the aggregate totals. Further research should examine the age- and sex-specific patterns to see if there are important differences in the migration and retention patterns. The patterns presented in Fig. 10 suggests that males and females have similar retention expectancies but with a couple of exceptions (e.g. higher retention levels for females born in India living in most area groupings). Individual characteristics could also be explored to determine the relative importance economic factors and social networks (Kritz et al. 2011). Moreover, we focused on the largest migrant populations. Similar analyses could be conducted for other migrants groups. Here, policies might have greater influence especially for relatively new and emerging immigrant groups.

In conclusion, the contributions of this research include a richer understanding of subsequent migration, which is an important part of the overall immigration experience. It seems obvious but not all migrants stay in the location they first move to. Some return to their origin or move to another country, and some relocate within the country. Australia is unique in comparison to other countries receiving large amounts of immigration in that many migrants enter on visas that meant to settle them in areas outside capital city areas where labour and particular skills are needed. Many of these communities are looking to international migration to sustain them and help them grow. The analysis presented in this paper shows that retention of both Australian-born and immigrant populations is a major obstacle to this need.

## Appendix 1

See Table 4.

**Table 4:** List of Australian area codes and area names

State	Area code	Area name	State	Area code	Area name
NSW	101	Sydney	WA	501	Perth
	102	Hunter		502	South West
	103	Illawarra		503	Lower Southern WA
	104	Mid-North Coast		504	Upper Southern WA
	105	North West NSW		505	Midlands
	106	Central West NSW		506	South Eastern WA
	107	Murrumbidgee		507	Central WA
	108	Murray		508	Pilbara
	109	West NSW		509	Kimberley
VIC	201	Melbourne	TAS	601	Greater Hobart
	202	Barwon		602	Northern Tasmania
	203	Western District		603	Mersey-Lyell
	204	Central Highlands	NT	701	Darwin
	205	Wimmera		702	Northern Territory
	206	Mallee	ACT	801	Canberra
	207	Loddon and Goulburn			
	208	Ovens-Murray			
	209	Gippsland			
QLD	301	Brisbane			
	302	Wide Bay-Burnett and Fitzroy			
	303	Darling Downs			
	304	South West Queensland			
	305	Central West Queensland			
	306	Mackay and Northern			
	307	Far North			
	308	North West			
SA	401	Adelaide			
	402	Yorke and Lower North			
	403	Murray Lands			
	404	South East			
	405	Eyre			
	406	Northern SA			

*NSW* New South Wales, *VIC* Victoria, *QLD* Queensland, *SA* South Australia, *WA* Western Australia, *TAS* Tasmania, *NT* Northern Territory, and *ACT* Australian Capital Territory

## Footnotes

1 This study on comparable internal migration indicators has been extended to include many more countries throughout the world in numerous recent papers as part of the IMAGE project led by Bell at the University of Queensland (see, e.g., Bell et al. 2015).
